# Superior mesenteric artery syndrome: An unusual cause of abdominal compartment syndrome and bilateral lower limb ischemia

**DOI:** 10.1259/bjrcr.20210174

**Published:** 2022-11-01

**Authors:** Avelyn EY Aw, James WK Lee, Ian JW Tan, Celene WQ Ng

**Affiliations:** 1 Yong Loo Lin School of Medicine, National University of Singapore, Singapore, Singapore; 2 Department of Surgery, Alexandra Hospital, Singapore, Singapore; 3 University Surgical Cluster, National University Hospital, Singapore, Singapore

## Abstract

Superior mesenteric artery (SMA) syndrome is a rare, unusual cause of proximal intestinal obstruction. It is characterized by compression of the third part of the duodenum secondary to narrowing of the anatomical space between the SMA and the aorta due to a loss of the intervening mesenteric fat pad.

This case highlights the challenge in obtaining a pre-operative radiological diagnosis in an extreme case of gastric outlet obstruction in SMA syndrome, fatally complicated by ACS and bilateral lower limb ischemia. It demonstrates that SMA syndrome remains important to exclude especially in patients with rapid weight loss and cardinal symptoms of intestinal obstruction, often requiring a high index of clinical suspicion.

## Clinical presentation

A 26-year-old cachectic Chinese female with anorexia nervosa and history of pathological depression presented to the Emergency Department with a sudden onset of generalized and progressive abdominal pain. This was associated with gross distension, generalized tenderness, and board-like rigidity. On presentation, she weighed 27.2 Kg, with a body mass index (BMI) of 9.71 Kg/m^2^. She was dehydrated, and unable to tolerate even sips of water.

Both lower limbs were Rutherford Stage III (irreversible ischemia). Bilateral femoral pulses were absent and without flow on Doppler ultrasound. Neurological and motor assessment demonstrated complete anesthesia bilaterally and power of 0/5 and 3/5 on the right and left lower limbs, respectively.

## Investigations and imaging findings

Significantly, hematological investigations showed a raised total white cell count of 17.9 × 10^9^/l (Neutrophils 93%), acute renal impairment with elevated levels of creatinine (140 mmol l^−1^) and urea (27 mmol l^−1^), accompanied by severe high-anion gap metabolic acidosis (HAGMA) with a pH of 7.02, base excess of −19.5 mmol l^−1^ and lactemia of 16.5 mmol l^−1^.

**Figure 1.**
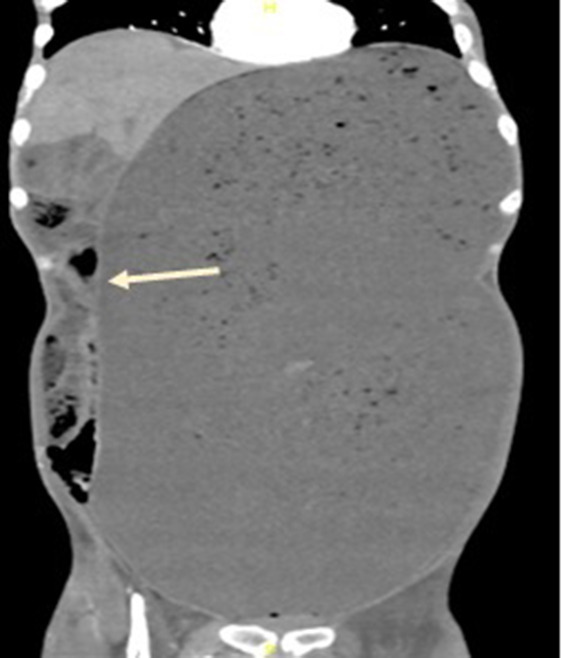
Gross gastric distension extending from the left upper quadrant to the pelvis, displacing the small bowel loops to the right.

An erect chest X-ray revealed a grossly distended stomach with no pneumoperitoneum, confirmed by a computed tomography (CT) scan of the abdomen and pelvis with intravenous contrast demonstrating gastric distension extending from the left upper quadrant to the pelvis, severely compressing the small bowel loops. (Figure 1).

**Figure 2.**
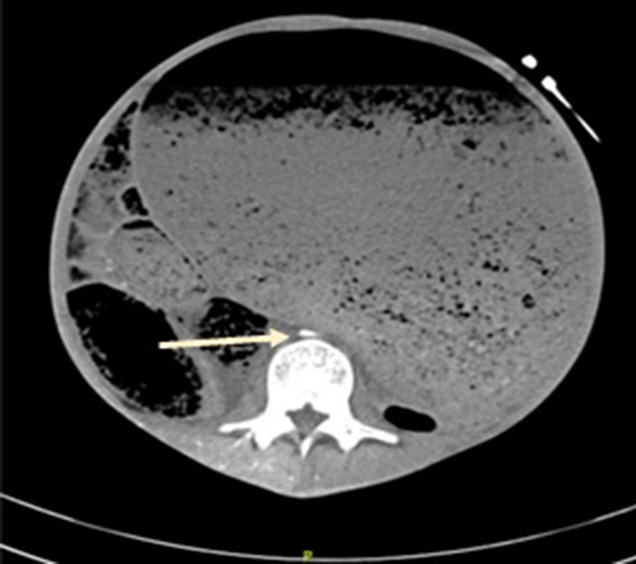
Compression of the abdominal aorta to a small “slit”. The IVC was unable to be visualized due to compression.

**Figure 3.**
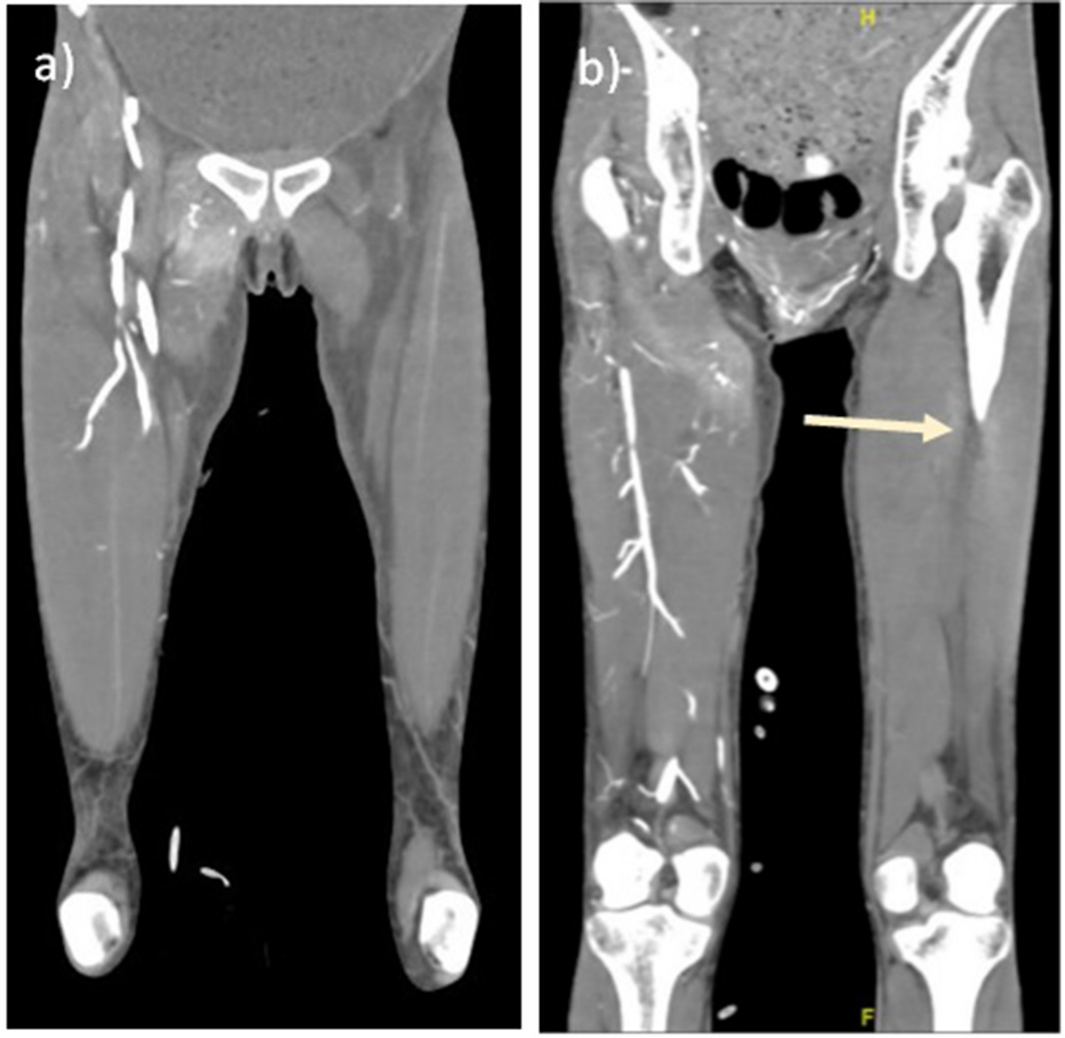
Right common femoral artery is enhancing to the level of the right popliteal artery, but is completely absent on the left.

This was complicated by ACS with radiological features including: hypoperfusion of the liver, spleen and kidneys; severe aortic compression and inability to visualize the inferior vena cava (IVC) due to increased abdominal compartment pressures (Figure 2); gastric compression on the origin of the left common iliac resulting in non-enhancement of distal vessels (Figure 3a); and enhancement of the right common femoral artery till the level of the popliteal artery (Figure 3b).

## Treatment

Our patient was immediately resuscitated as per the working diagnosis of gastric outlet obstruction (GOO) - insertion of large-bore nasogastric tube for decompression and a urinary catheter, with administration of broad-spectrum intravenous antibiotics. She had immediate decompressive laparotomy, revealing a grossly distended and ischemic stomach with local perforation extending from the xiphisternum to the pelvis, and duodenal dilatation with a transition point at the third portion (D3).

A gastrostomy drained 15 liters of undigested, decomposing food content. Immediate reperfusion of bilateral lower limbs followed, with the femoral pulses felt bilaterally. Following temporary abdominal closure and in view of the severe and prolonged acute limb ischemia and swelling, leg and thigh fasciotomies were indicated for prophylaxis against the development of acute lower limb compartment syndrome after revascularization.

## Outcome

Despite these measures, she eventually succumbed to multisystemic dysfunction with gastrointestinal, renal and cardiovascular compromise and deceased.

## Discussion

Classically, SMA syndrome is diagnosed based on radiological imaging criteria^1^:

1. Duodenal obstruction with an abrupt cutoff in D3
2. Aorto-SMA angle of ≤25° (most sensitive)

The normal aorto-SMA angle ranges between 35° and 65° and correlates with the patient’s BMI.^2^ As such, significant and rapid weight loss remains as the most common cause of SMA syndrome. In our patient, her weight loss was attributed to anorexia nervosa, but weight loss in SMA syndrome is also commonly associated with other medical, surgical, or psychological conditions.^3^ As the presentation of symptoms tends to arise insidiously and in progressive severity, diagnosis is often delayed and patients present in states of complications including electrolyte derangements, severe metabolic acidosis, gastric outlet, and intestinal obstruction, or ischemic perforation, which in this case proved to be fatal.

**Figure 4.**
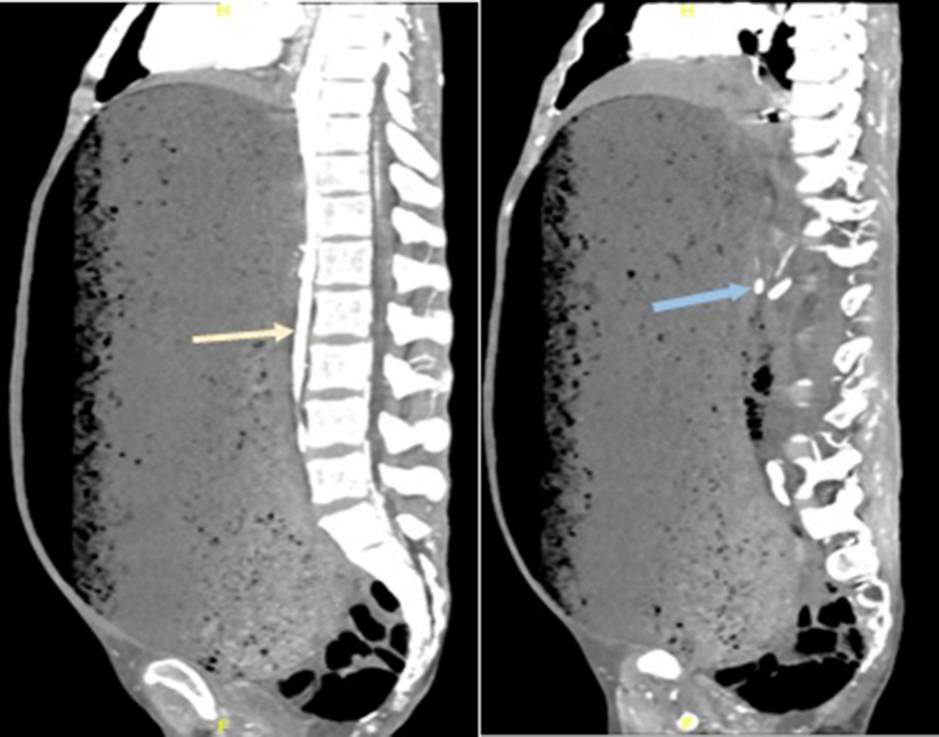
Severe gastric distension and anatomical distortion resulting in difficulty visualizing classical diagnostic features of SMA syndrome on a lateral angiography. Aorta (yellow arrow), SMA (blue arrow)

In our patient, the severe gastric distension and anatomical distortion with displacement of the duodeno-jejunal junction to the right hemi-abdomen made radiological diagnosis of classical SMA syndrome on a lateral CT angiography unfeasible (Figure 4). The diagnosis and cause of death were subsequently confirmed by the state coroner to be gastric outlet obstruction secondary to SMA syndrome.

To our knowledge, only one other case presents complications of similar severity; however, the patient was diagnosed with SMA syndrome prior to the acute deterioration.^4^ Our case unusually highlights the challenge in obtaining a pre-operative radiological diagnosis. While a CT angiography was done, additional multiplanar reconstructions and 3D rendering would be critical in diagnosing as well.^5^

During this admission, our patient was treated on the clinical diagnosis of GOO and complications of ACS. There were multiple missed GOO diagnosis prior to this admission, as her severe weight loss, intolerance for food and liquids, and progressive symptoms of obstruction and gastric distension were attributed to her history of anorexia and thus repeatedly dismissed. Inevitably, her severe and decompensated presentation was indicative of end-stage disease.

SMA syndrome remains important to exclude especially in patients with rapid weight loss and cardinal symptoms of intestinal obstruction,^6^ often requiring a high index of clinical suspicion.^7^

Another entity resulting from a similar anatomical abnormality of a narrowed aortomesenteric angle resulting in overlying compression is Nutcracker syndrome, where the SMA compresses on the left renal vein instead of the duodenum.

Principles of conservative treatment in SMA syndrome include decompression and encouraging weight gain via supplemental nutrition, considering underlying medical or psychiatric disorders contributing to significant weight loss.^8^ Although, this is more effective in acute cases and prior to pathological duodenal structural changes.^7^

Surgical intervention is indicated in failure of conservative management, with the main options being Strong’s procedure, gastrojejunostomy or duodenojejunostomy.^9^ However, in acute episodes with severe presentations like our case, acute management principles of an acute surgical abdomen with damage control surgery take precedence over such definitive surgery approaches initially.

## Learning points

- Although rare, early recognition of SMA syndrome as a possible diagnosis and understanding of its relevant complications is crucial.
- Highlighting early recognition of ACS and its appropriate management.
- There is a need to consider and exclude SMA syndrome as a diagnosis in patients with anorexia nervosa presenting with a history of recurrent nausea and vomiting.
